# Deficiency of GntR Family Regulator MSMEG_5174 Promotes *Mycobacterium smegmatis* Resistance to Aminoglycosides via Manipulating Purine Metabolism

**DOI:** 10.3389/fmicb.2022.919538

**Published:** 2022-07-11

**Authors:** Wanyan Deng, Zengzhang Zheng, Yi Chen, Maoyi Yang, Jun Yan, Wu Li, Jie Zeng, Jianping Xie, Sitang Gong, Huasong Zeng

**Affiliations:** ^1^The Joint Center for Infection and Immunity, Guangzhou Institute of Pediatrics, Guangzhou Women and Children’s Medical Center, Guangzhou, China; ^2^Institut Pasteur of Shanghai, Chinese Academy of Sciences, Shanghai, China; ^3^Key Laboratory of Molecular Biology for Infectious Diseases (Ministry of Education), Department of Infectious Diseases, Institute for Viral Hepatitis, The Second Affiliated Hospital of Chongqing Medical University, Chongqing, China; ^4^Department of Respiratory Medicine, The First People’s Hospital of Yunnan Province, Kunming, China; ^5^Affiliated Hospital of Kunming University of Science and Technology, Kunming, China; ^6^State Key Laboratory Breeding Base of Eco-Environment and Bio-Resource of the Three Gorges Area, Key Laboratory of Eco-Environments in Three Gorges Reservoir Region, Ministry of Education, School of Life Sciences, Institute of Modern Biopharmaceuticals, Southwest University, Chongqing, China

**Keywords:** *Mycobacterium smegmatis*, GntR, MSMEG_5174, purine metabolism, aminoglycoside antibiotics resistance

## Abstract

The increasing incidence of drug-resistant tuberculosis is still an emergency for global public health and a major obstacle to tuberculosis treatment. Therefore, deciphering the novel mechanisms of *mycobacterial* antibiotic resistance is crucial for combatting the rapid emergence of drug-resistant strains. In this study, we identified an unexpected role of *Mycobacterium smegmatis* GntR family transcriptional regulator MSMEG_5174 and its homologous gene *Mycobacterium tuberculosis* Rv1152 in aminoglycoside antibiotic resistance. Deficiency of MSMEG_5174 rendered *Mycobacterium smegmatis* highly resistant to aminoglycoside antibiotic treatment, and ectopic expression of Rv1152 in MSMEG_5174 mutants restored antibiotic-induced bacterial killing. We further demonstrated that MSMEG_5174 negatively regulates the expression of purine metabolism-related genes and the accumulation of purine metabolites. Moreover, overexpression of xanthine dehydrogenase MSMEG_0871 or xanthine treatment elicited a significant decrease in aminoglycoside antibiotic lethality for *Mycobacterium smegmatis*. Together, our findings revealed MSMEG_5174 as a metabolic regulator and hint toward unexplored crosstalk between purine metabolism and antibiotic resistance.

## Introduction

Tuberculosis (TB), an infectious disease caused by *Mycobacterium tuberculosis* (*M. tuberculosis*), is the leading cause of death worldwide. In 2020, an estimated 10 million people developed active TB and approximately 1.5 million individuals died from TB (Jeremiah et al., 2022). The emergence of multidrug-resistant and extensively drug-resistant strains highlights an urgency for novel strategies to protect against TB (Nahid et al., 2016; Jeremiah et al., 2022). Generally, antibiotics inhibit bacterial growth mainly by targeting bacterial replication and protein synthesis (Walsh, 2000). In turn, bacterial physiological processes, including gene transcription and protein translation, stress responses, and DNA repair, have been reported to be associated with the lethality of antibiotics (Dwyer et al., 2015; Gruber and Walker, 2018; Yang et al., 2018). Interestingly, increasing evidence has proved that bacterial metabolism plays a key role in antibiotic-mediated killing (Allison et al., 2011; Cho et al., 2014; Peng et al., 2015; Meylan et al., 2017; Fan et al., 2018). Therefore, it is crucial to understand how bacteria manipulate metabolism to interfere with antibiotic-mediated lethality.

The GntR family transcriptional regulators, originally identified in *Bacillus subtilis* (*B. subtilis*), are named after a gluconate operon repressor (Fujita et al., 1986). These family regulators are widespread in bacteria and possess a conserved N-terminal DNA-binding domain and a C-terminal domain with the variable in length and structure (Suvorova et al., 2015). Based on the characteristics of the C-terminal domain, GntR family regulators are further classified into six subfamilies, including FadR, MocR, HutC, YtrA, AraA, and PlmA subfamily (Rigali et al., 2002; Suvorova et al., 2015). The YtrA subfamily is the least representative GntR-like transcription factor in the bacterial genomes (Suvorova et al., 2015). MSMEG_5174 is the only GntR regulator found in *Mycobacterium smegmatis* (*M. smegmatis*) with the signatures of the YtrA subfamily (Rigali et al., 2002). Our previous study revealed *M. tuberculosis* GntR family regulator Rv1152, which is a homolog of *M. smegmatis* MSMEG_5174, inhibits the expression of genes involved in vancomycin resistance (Zeng et al., 2016). *B. subtilis* GntR subfamily repressor YtrA regulates an operon associated with acetoin utilization (Yoshida et al., 2000) and is responsive to cell wall antibiotics (Salzberg et al., 2011). It would be interesting to investigate whether MSMEG_5174 modulates such an operon associated with bacterial metabolism and antibiotic resistance. Herein, we demonstrate an unexpected and exciting role of *M. smegmatis* MSMEG_5174 and its homologous gene *M. tuberculosis* Rv1152 in modulating purine metabolism and aminoglycoside antibiotic resistance.

## Materials and Methods

### Reagents

Xanthine (X8030) was purchased from Solarbio Life Science, Beijing, China. Middlebrook 7H9 Broth (271310) was purchased from BD/Difco, Franklin Lakes, NJ, United States. Amikacin (A602232), kanamycin (A100408), gentamicin (A100304), streptomycin (A100382), chloramphenicol (A100230), ciprofloxacin (A600310), rifampicin (A600812), and isoniazid (A600544) were purchased from Sangon Biotech, Shanghai, China. The Anti-His antibody (#9991) was purchased from Cell Signaling Technology, Danvers, MA, United States.

### Strains and Growth Conditions

*Mycobacterium smegmatis* mc^2^ 155 WT, MSMEG_5174 gene knock-out strain (ΔMSMEG_5174), and gene complementary strain (ΔMSMEG_5174 + pRv1152) were kindly gifts provided by Prof. Jianping Xie (Southwest University, Chongqing, China). The full length of the xanthine dehydrogenase encoding gene MSMEG_0871 was amplified from *M. smegmatis* mc^2^ 155 genomic DNA using gene-specific primers pALACE-MSMEG_0871 (F and R) listed in Table 1. The resulting PCR products were digested and inserted into pALACE to generate the pALACE-MSMEG_0871 plasmid. Then, pALACE and pALACE-MSMEG_0871 were electroporated into *M. smegmatis*. The resulting strains harboring pALACE and pALACE-MSMEG_0871 were named MS_Vec and MS_MSMEG_0871, respectively. The expression of MSMEG_0871 was detected by Western blotting using an anti-His antibody. All the strains were grown in 7H9 broth or 7H9 agar supplemented with 0.5% glycerol, 0.05% Tween 80, and 0.2% glucose (Goude and Parish, 2008). Acetamide and hygromycin were added when needed.

**TABLE 1:** Primers used in the study.

Primer	Sequence (5′–3′)
MSMEG_1135 (F)	GCTACCGCGTCATCCAGA
MSMEG_1135 (R)	TCAGTCGCATTTGAGGTC
MSMEG_0869 (F)	GGAGGTTGATGGCGAGTT
MSMEG_0869 (R)	GAGAAATGTGGCGAAGCA
MSMEG_0870 (F)	AGGTTCTCGATGCATTCTTT
MSMEG_0870 (R)	AGGTAGTCGGACATGTTGG
MSMEG_0871 (F)	GGTGGCGCTCGACATACA
MSMEG_0871 (R)	GCGATGGTCTCGAGCTCA
MSMEG_0872 (F)	ACACACGAAACGCACGACA
MSMEG_0872 (R)	TTCACGCAGCATGTCCAGC
MSMEG_0873 (F)	ATGTTGTTTTCACCCGGT
MSMEG_0873 (R)	TTGTGATGCAGCGTGATT
pALACE-MSMEG_0871 (F)	GGAATTCGTGCATCCGTTCGC
pALACE-MSMEG_0871 (R)	CGGATCCACATTGCACACCCG

### Growth Curve Measurement

The overnight cultures of WT, ΔMSMEG_5174, MS_Vec and MS_MSMEG_0871 strains were reinoculated in a fresh 7H9 medium at the ratio of 1:1,000 dilution. Each strain was incubated at 37°C with shaking through the entire growth phase. Samples were collected at the same growth stage, and the OD_600_ values were measured every 3 h after growth initiation. Experiments were performed in triplicates, and the average values were used to generate growth curves.

### Scanning Electron Microscopy Analysis

The WT and ΔMSMEG_5174 were grown into a logarithmic phase and subjected to scanning electron microscopy (SEM) analysis according to the previously described method with minor modification (Lv et al., 2014). Generally, bacterial pellets were collected and fixed with 2.5% glutaraldehyde. Then, the samples were dehydrated using a series of ethanol grades and subjected to SEM (FEI Quanta 200, Hillsbor, OR, United States) after lyophilization and gold coating. The morphology of each strain was observed, and the length of the bacteria was measured.

### Transmission Electron Microscopy Analysis

Logarithmic phase bacterial samples were prepared according to the previously described with minor modifications (Lv et al., 2014; Chuang et al., 2015). Briefly, the samples were fixed with 2.5% glutaraldehyde and post-fixed with 1% osmium tetroxide, followed by dehydration. At room temperature, the samples were transferred to a 1:1 mixture of absolute acetone and epoxy resin for 1 h after being placed in absolute acetone for 20 min. Then the samples were transferred to a 1:3 mixture of absolute acetone and epoxy resin for 3 h, followed by transferring to pure epoxy resin overnight. Ultrathin sections, obtained by using an ultramicrotome, were post-stained with uranyl acetate and lead citrate. Specimens were observed by transmission electron microscopy (TEM) (Hitachi H-7650, Japan).

### Minimum Inhibitory Concentration Determinations for Antibiotics

Aminoglycoside antibiotics including gentamicin, streptomycin, kanamycin, and amikacin were used in this study. The minimum inhibitory concentration (MIC) of these antibiotics for WT and ΔMSMEG_5174 were measured according to the method described previously (Zeng et al., 2016). The dilution of all antibiotics was performed in 96-well plates. A twofold dilution of each antibiotic was prepared in the test wells. The final concentration range of these antibiotics was as follows: gentamicin (0.25–32 μg/ml), streptomycin (0.03125–4 μg/ml), kanamycin (0.25–32 μg/ml), and amikacin (0.1–12.8 μg/ml). The prepared strains were added and the plates were incubated at 37°C for 3 days. Visual inspection of the size of the bacterial pellets was used for the MIC determination. MIC was determined as the lowest concentration of antibiotic when the bacterial activity was killed at least 99% in liquid medium.

### Antibiotic Lethality Assays

Antibiotic lethality assay was performed as previously described (Zeng et al., 2016). *M. smegmatis* strains, including MS_Vec, MS_MSMEG_0871, WT, ΔMSMEG_5174, and ΔMSMEG_5174 + pRv1152, were grown into logarithmic phase. The bacterial cells were collected and diluted in a 7H9 medium to an OD_600_ of 0.1. The final concentrations of all indicated antibiotics were as follows: gentamicin (1, 2, 4, 8, 16, and 32 μg/ml), kanamycin (0.25, 0.5, 1, 2, 4, and 8 μg/ml), streptomycin (0.03125, 0.0625, 0.125, 0.25, 0.5, 1, and 2 μg/ml), amikacin (0.1, 0.2, 0.4, 0.8, 1.6, and 3.2 μg/ml), ciprofloxacin (2.5, 5, 10, 20, and 40 μg/ml), chloramphenicol (320, 640, 1,280, 2,560, and 5,120 μg/ml), rifampicin (80, 160, 320, 640, and 1,280 μg/ml), and isoniazid (20, 40, 80, 160, and 320 μg/ml). For metabolite supplementation experiments, bacterial cells were grown in a 7H9 medium supplied with 1 mM xanthine and subjected to an antibiotic killing assay. After indicated antibiotic treatment, 100 μl aliquot samples were removed for 10-fold serial dilution, and the diluted bacterial cells were plated 10 μl into 7H9 agar plates. All plates were incubated at 37°C for 3 days and the colony-forming units (c.f.u) were counted. Percent survival was calculated by dividing the c.f.u of treated groups by that of the control group.

### Transcriptome Assay and Data Analysis

Logarithmic phase WT and ΔMSMEG_5174 strains were subjected to transcriptome analysis. The bacteria pellets were collected and total RNA was isolated using the RNeasy mini kit (Qiagen, Germany). Libraries were constructed and subjected to sequencing using the Illumina HiSeq 2500 (Illumina, United States) at Shanghai Biotechnology Corporation. The raw reads were preprocessed and low-quality reads were filtered out. The fold change of each gene was estimated according to the FPKM value generated by Cufflinks v2.1.1 after genome mapping. The Cuffdiff and false discovery rate (FDR) were used for dysregulated gene identification and multiple testing correction, respectively. The dysregulated genes were selected and filtered by FDR ≤ 0.05 and fold-change ≥ 2. The raw data were deposited to NCBI and the accession numbers for WT and ΔMSMEG_5174 are SRR19667998 and SRR19667999, respectively.

### qRT-PCR Analysis

The total RNA was isolated using the RNeasy mini kit (Qiagen, Germany). cDNA was synthetized from 1 μg of total RNA using the RevertAid First Strand cDNA Synthesis kit (Thermo Fisher, United States) with random primers. Quantitative real-time PCR was performed by using the iQ SYBR Green Supermix in the CFX96 Touch System (Bio-Rad, United States) under the following thermocycling parameters: 95°C for 5 min and 40 cycles at 95°C for 30 s, 60°C for 30 s and 72°C for 30 s. Gene expression was normalized to *sig*A (Wang et al., 2011) and gene-specific primers are listed in Table 1.

### Metabolome Assay and Data Analysis

Sample preparation and analysis were performed as previously described with minor modifications (Chuang et al., 2015). WT, ΔMSMEG_5174 and ΔMSMEG_5174 + pRv1152 were grown into logarithmic phase. Bacterial pellets, including eight replicates of each sample, were collected and subjected to μHPLC (1290 Infinity LC, Agilent Technologies, Santa Clara, CA, United States) coupled to a quadrupole time-of-flight (AB Sciex TripleTOF 6600, United States) analysis (Ivanisevic et al., 2013). The ProteoWizard MSConvert tool was used to convert the raw MS data into the MzXML files that can be processed using the XCMS for data analysis (Benton et al., 2008). The metabolites were identified by automated comparison of the ion features in the experimental samples to a reference library of chemical standards. After Pareto scaling, principal component analysis (PCA) and partial least-squares-discriminant analysis (PLS-DA) were performed, respectively. Metabolites were determined by the combination of variable importance in project (VIP) value and a two-tailed Student’s test (*p*-value). The metabolites with a significant difference were determined and filtered by the VIP values ≥ 1.0 and *p*-value ≤ 0.1. The dysregulated metabolites were subjected to Kyoto Encyclopedia of Genes and Genomes (KEGG) pathway analysis.

### Ethidium Bromide Accumulation Assay

The accumulation of ethidium bromide (EB) was measured as previously described (Chuang et al., 2015; Deng et al., 2020). Logarithmic phase bacterial cells cultured in the presence or the absence of xanthine were collected and washed twice with PBS containing 0.05% Tween 80, and the resulting bacterial cells were subjected to EB accumulation assays. The time-course fluorescence intensity was detected by excitation at 544 nm and emission at 590 nm using Biotek Synergy H1 (Winooski, VT, United States). All data were normalized to the first-time point reading of each well.

### Statistical Analysis

All statistics were calculated using GraphPad Prism 8 software and Student’s *t*-test was used to determine the statistical significance between different groups. ^∗∗∗^*P* < 0.001, ^∗∗^*P* < 0.01, ^∗^*P* < 0.05, means ± SD from at least three biological replicates. Data are representative of at least three independent experiments.

## Results

### Deletion of MSMEG_5174 Has no Effect on the Basic Characteristics of *M. smegmatis*

**FIGURE 1:**
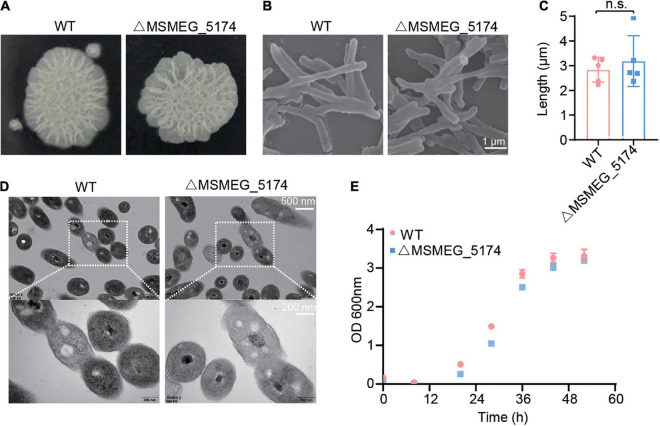
The effect of MSMEG_5174 deficiency on the basic characteristics of *Mycobacterium smegmatis*. **(A)** The morphology of WT and MSMEG_5174 mutants grown in 7H9 agar. **(B)** Scanning electron microscopic micrographs of WT and MSMEG_5174 mutants. **(C)** The length of WT and MSMEG_5174 mutants, each point represents a single cell from a field of view. **(D)** Transmission electron microscopic micrographs of WT and MSMEG_5174 mutants. **(E)** The growth of WT and MSMEG_5174 mutants.

To test the effect of GntR family regulator MSMEG_5174 deficiency on bacteria itself, the morphology and structure of *M. smegmatis* (WT) and MSMEG_5174 mutants (ΔMSMEG_5174) were monitored. No significant difference was detected in morphology between WT and ΔMSMEG_5174 strains grown in 7H9 agar (Figure 1A). SEM analysis revealed a normal rod morphology and similar bacterial length in MSMEG_5174 mutants compared to WT strains (Figures 1B,C). In addition, TEM analysis also showed that MSMEG_5174 deletion has no effect on bacterial membrane integrity and intracellular structures (Figure 1D). Moreover, the growth of MSMEG_5174 mutants was similar to that of WT strains (Figure 1E). Taken together, GntR family regulator MSMEG_5174 deficiency has no effect on the growth, morphology, and intracellular structure of the *M. smegmatis*.

### MSMEG_5174 Negatively Regulates Genes Associated With Purine Metabolism

**FIGURE 2:**
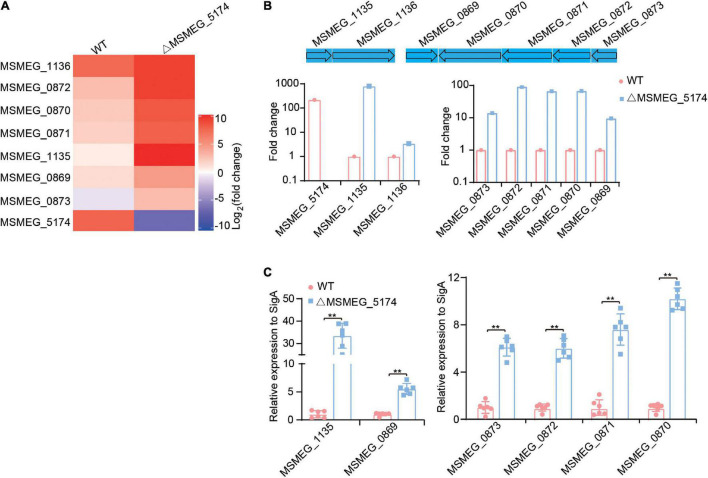
Transcriptome profiling of WT and MSMEG_5174 mutants. **(A)** Heat maps of dysregulated genes in WT and MSMEG_5174 mutants. **(B)** The fold changes of gene expression in MSMEG_5174 mutants calculated and normalized to WT strains. **(C)** qRT-PCR was used to verify the expression of the genes in WT and MSMEG_5174 mutants. **P < 0.01.

Studies have shown that GntR regulators regulate genes associated with metabolic processes, including carbon metabolism in *Salmonella typhimurium* (Titgemeyer et al., 1995), amino acid metabolism in *Pseudomonas putida* (Allison and Phillips, 1990), and glucose metabolism in *Pseudomonas aeruginosa* (Daddaoua et al., 2017). To detect whether *M. smegmatis* MSMEG_5174 regulates the expression of metabolism-related genes, WT and MSMEG_5174 mutants were subjected to transcriptome analysis. Two operons, in which all genes were significantly upregulated in MSMEG_5174 mutants, attracted our attention. One operon contains two hypothetical proteins, MSMEG_1135 and MSMEG_1136, which were approximately 799- and 3.4-fold upregulated in MSMEG_5174 mutants, respectively (Figures 2A,B). The other operon contains genes potentially associated with bacterial purine metabolism. These genes are oxidoreductase MSMEG_0870 (67-fold), putative xanthine dehydrogenase MSMEG_0871 (66-fold), twin-arginine translocation pathway signal protein MSMEG_0872 (90-fold), and hypothetical protein MSMEG_0873 (14-fold) (Figures 2A,B). In addition, a hypothetical protein MSMEG_0869 near this operon was also upregulated about 7.5-fold in MSMEG_5174 mutants (Figures 2A,B). To verify the expression of these dysregulated genes in WT and MSMEG_5174 mutants, the total RNA of each strain was extracted and subjected to qRT-PCR. Consistent with transcriptome data, the expression of these genes was significantly upregulated in ΔMSMEG_5174 strains compared to WT strains (Figure 2C). These data demonstrate that MSMEG_5174 negatively regulates the expression of genes associated with purine metabolism and genes with unknown functions.

### MSMEG_5174 Deficiency Contributes to Antibiotic Resistance via Purine Metabolism

**FIGURE 3:**
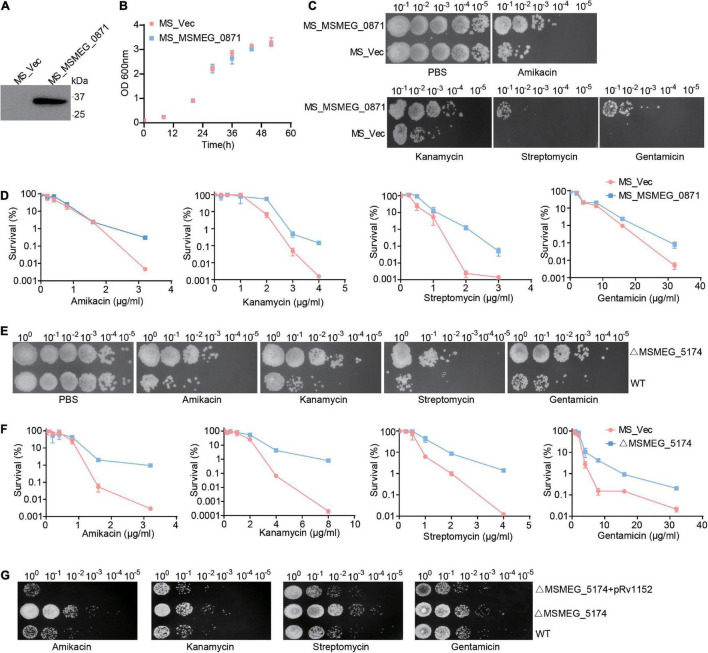
MSMEG_5174 deficiency blocks aminoglycoside antibiotics mediated bacterial killing. **(A)** The exogenous expression of MSMEG_0871 in *M. smegmatis*. **(B)** The growth of MS_Vec and MS_MSMEG_0871. **(C)** MS_Vec and MS_MSMEG_0871 were treated with indicated concentration of aminoglycoside antibiotics. **(D)** MS_Vec and MS_MSMEG_0871 were treated with different concentration of aminoglycoside antibiotics. **(E)** WT and ΔMSMEG_5174 were subjected to indicated concentration of aminoglycoside antibiotics treatment. **(F)** WT and ΔMSMEG_5174 were treated with different concentration of aminoglycoside antibiotics. **(G)** WT, ΔMSMEG_5174 and ΔMSMEG_5174 + pRv1152 strains were treated with indicated concentration of aminoglycoside antibiotics.

Yang et al. (2019) demonstrated that *Escherichia coli* mutants deficient in genes involved in the early steps of purine biosynthesis exhibit a significant increase in gentamicin lethality compared to the WT, indicating a role of purine metabolism in aminoglycoside lethality. We reasoned that up-regulation of purine metabolism-related genes in MSMEG_5174 mutants may contribute to the decreased aminoglycoside antibiotic lethality. To this end, we overexpressed the xanthine dehydrogenase gene MSMEG_0871 in *M. smegmatis* and monitored aminoglycoside antibiotics-mediated bacterial killing. Overexpression of MSMEG_0871 had no impact on the growth of *M. smegmatis* (Figures 3A,B). Strikingly, *M. smegmatis* overexpressing MSMEG_0871 exhibited a significant increase in survival in aminoglycoside antibiotics-mediated killing compared to the control group (MS_Vec) (Figures 3C,D), indicating that MSMEG_0871 facilitates aminoglycoside antibiotic resistance of *M. smegmatis*. In support of this, MSMEG_5174 deficiency also reduced bacterial killing by aminoglycoside antibiotics treatment (Figures 3E,F). In contrast, there was no significant difference in survival between WT and MSMEG_5174 mutants when treated with ciprofloxacin, chloramphenicol, rifampicin, or isoniazid (Supplementary Figure 1). These data suggest that MSMEG_5174 deletion has specificity and high resistance to aminoglycoside antibiotics. Moreover, the MIC for both amikacin and kanamycin increased by 4-fold, and the MIC for gentamicin and streptomycin increased by 2- and 16-fold in MSMEG_5174 mutants compared to WT, respectively (Table 2). To further confirm the function of MSMEG_5174 in regulating antibiotics resistance, we introduced an MSMEG_5174 homologs Rv1152 from *M. tuberculosis* into the MSMEG_5174 mutants and found it could rescue the susceptibility of MSMEG_5174 mutants to aminoglycoside antibiotics (Figure 3G). These data suggest that purine metabolism-associated genes controlled by *M. smegmatis* MSMEG_5174 or its homologs Rv1152 in *M. tuberculosis* play an important role in aminoglycoside antibiotic resistance.

**TABLE 2:** The MIC of WT and ΔMSMEG_5174 to antibiotics.

Antibiotics (μg/ml)	WT	MSMEG_5174
Amikacin	0.25	1
Kanamycin	1	4
Streptomycin	0.125	2
Gentamicin	2	4

### Purine Metabolites Accumulate in MSMEG_5174 Mutants

**FIGURE 4:**
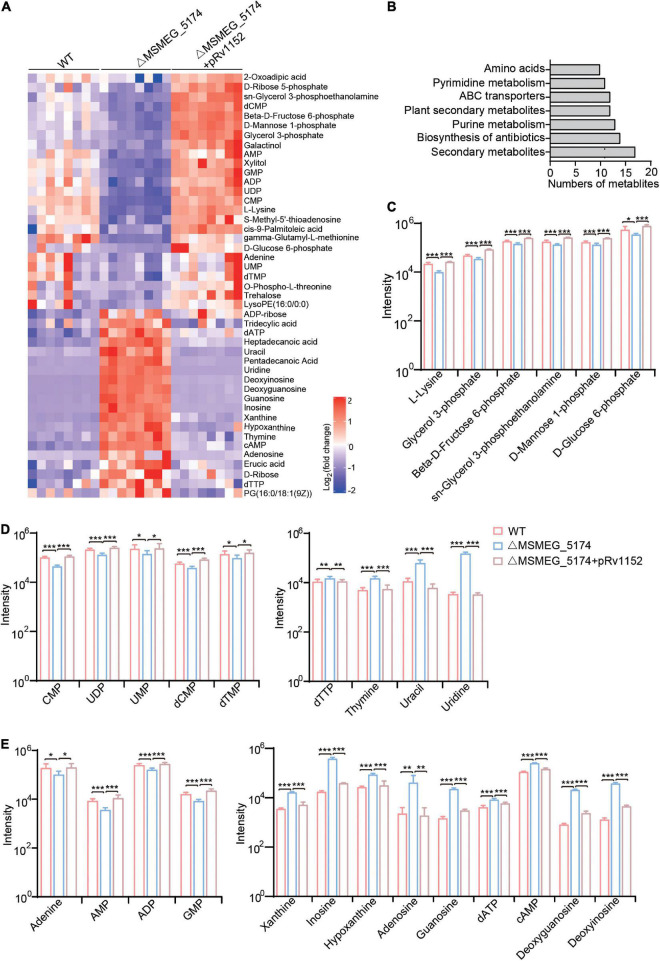
Metabolic profiles of WT, ΔMSMEG_5174 and ΔMSMEG_5174 + pRv1152. **(A)** Heat map of dysregulated metabolites in WT, ΔMSMEG_5174 and ΔMSMEG_5174 + pRv1152 strains. Map scale (blue to red: low to high abundance). **(B)** Enriched KEGG pathways in MSMEG_5174 mutants. **(C)** Dysregulated amino acids and carbon source derived from secondary metabolism. **(D)** Dysregulated pyrimidine metabolites. **(E)** Dysregulated purine metabolites. ***P < 0.001, **P < 0.01, *P < 0.05.

To explore whether the MSMEG_5174 deletion modifies the metabolites associated with purine metabolism, metabolomic profiling of WT, ΔMSMEG_5174, and ΔMSMEG_5174 + pRv1152 strains with eight biological replicates are analyzed by LC-MS/MS. Hierarchical clustering was used to rank metabolites whose abundance differed significantly among these strains (Figure 4A). Compared with WT strains, seven strongly impacted KEGG pathways were identified in MSMEG_5174 mutants, including secondary metabolites, biosynthesis of antibiotics, purine metabolism, plant secondary metabolites, ABC transporters, pyrimidine metabolism, and amino acids (Figure 4B). Among these pathways, dysregulated metabolites in MSMEG_5174 mutants, including amino acids, carbon sources from secondary metabolites, pyrimidine metabolites, and purine metabolites, could be rescued by Rv1152 (Figures 4C–E). The abundance of one amino acid (L-lysine) and five carbon sources (glycerol 3-phosphate, β-D-Fructose 6-phosphate, sn-Glycerol 3-phosphoethanolamine, D-mannose 1-phosphate, and D-glucose 6-phosphate) were decreased in MSMEG_5174 mutants compared to WT (Figure 4C). For pyrimidine metabolites, four metabolites (dTTP, thymidine, uracil, and uridine) were accumulated and five metabolites (CMP, UDP, UMP, dCMP, and dTMP) were reduced in MSMEG_5174 mutants compared to WT (Figure 4D). Notably, compared with WT strains, nine purine metabolites including inosine, hypoxanthine, xanthine, adenosine, guanosine, dATP, cAMP, deoxyguanosine, and deoxyinosine were increased, while four purine metabolites (adenine, AMP, ADP, and GMP) were decreased in MSMEG_5174 mutants (Figure 4E). The dysregulation of metabolites from purine metabolism may be caused by the up-regulated expression of genes associated with purine metabolism in MSMEG_5174 mutants (Figure 2). Collectively, MSMEG_5174 in *M. smegmatis* or Rv1152 in *M. tuberculosis* regulates the expression of genes involved in purine metabolism, resulting in metabolites accumulation or reduction that may affect aminoglycoside antibiotics lethality.

### Xanthine Reduces Bacterial Cell Wall Permeability

**FIGURE 5:**
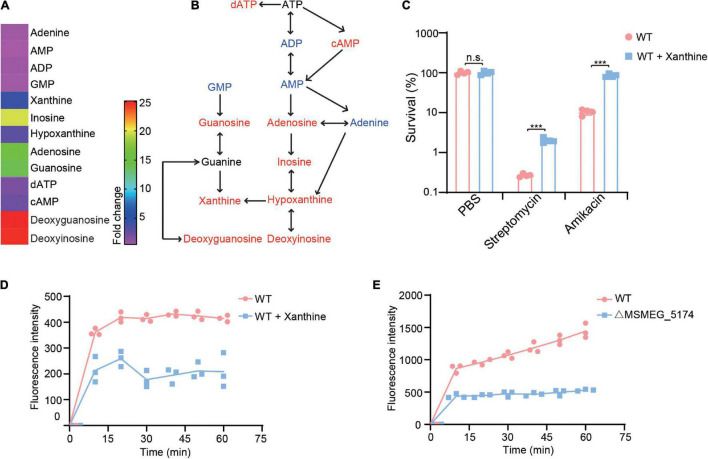
Xanthine decreases aminoglycoside antibiotics lethality for *M. smegmatis*. **(A)** Fold changes of purine metabolites in MSMEG_5174 mutants compared to WT. **(B)** Purine metabolic pathway. Red and blue color represent upregulated and downregulated metabolites in MSMEG_5174 mutants, respectively. **(C)** Percent survival of *M. smegmatis* in the presence or absence of xanthine with indicated antibiotics treatment. **(D)** EB accumulation in WT cultured with or without xanthine. **(E)** EB accumulation in WT and MSMEG_5174 mutants. **P < 0.01.

The enzyme xanthine oxidoreductase consists of two different forms including xanthine oxidase and xanthine dehydrogenase, which catalyzes the oxidation of hypoxanthine to xanthine and xanthine to uric acid in humans (Battelli et al., 2014). We identified *M. smegmatis* xanthine dehydrogenase MSMEG_0871 contributes to aminoglycoside antibiotics resistance (Figures 3A–D), which may be caused by xanthine accumulation during purine metabolism (Figure 4E). To explore whether xanthine accumulation could decrease aminoglycoside antibiotics against *M. smegmatis*, xanthine from purine metabolism was chosen and exogenously added for aminoglycoside antibiotics treatment (Figures 5A,B). WT strains were pre-treated with or without xanthine and then challenged with streptomycin or amikacin. As expected, the survival of *M. smegmatis* was significantly increased when treated with streptomycin or amikacin in the presence of xanthine (Figure 5C), suggesting that xanthine could enhance aminoglycoside antibiotics resistance of *M. smegmatis*. To further understand the potential role of xanthine in reducing antibiotics lethality, we investigated EB accumulation in WT strains treated with or without xanthine. Interestingly, WT strains showed significantly reduced EB accumulation when treated with xanthine (Figure 5D), suggesting that xanthine opaques the cell wall, leading to reduced antibiotics uptake. Similarly, MSMEG_5174 mutants showed lower uptake of the EB compared to WT strains (Figure 5E). These results indicate a role of MSMEG_5174 in the negatively regulation of purine metabolites accumulation, resulting in reduced antibiotics uptake and lethality.

## Discussion

Aminoglycosides, such as gentamicin, kanamycin, streptomycin, and amikacin, are important second-line anti-TB drugs via targeting the 30S subunit of the ribosome and interfering with translational fidelity (Purohit and Stern, 1994; Borovinskaya et al., 2007). Generally, aminoglycosides-resistant bacteria are typically caused by 16S rRNA modification, aminoglycoside modifying enzymes, and efflux pumps (Poole, 2005; Jana and Deb, 2006; Shakil et al., 2008; Garneau-Tsodikova and Labby, 2016). Recently, the altered bacterial metabolism was reported to be important to aminoglycoside antibiotics-mediated killing (Allison et al., 2011; Cho et al., 2014; Peng et al., 2015; Meylan et al., 2017; Yang et al., 2017; Fan et al., 2018). In view of the importance of metabolites in promoting or impairing antibiotics lethality, understanding of how bacterial metabolism interfaces with antibiotics efficacy has the potential to shed light on drug discovery (Murima et al., 2014; Bald et al., 2017).

The presence of a penetration barrier for antibiotics uptake in bacteria has been regarded as a major problem of antibiotics lethality (Lewis, 2013). Studies have shown that metabolites potentiate aminoglycoside antibiotics uptake and bactericidal activity via stimulating proton motive force (PMF) production (Allison et al., 2011; Peng et al., 2015; Meylan et al., 2017; Deng et al., 2020). Non-replicating *M. tuberculosis* displayed a reduction in antibiotics uptake and the phenotype of antibiotics resistance (Sarathy et al., 2013), however, the underlying mechanism keeps unknown. In this study, we identified the purine metabolism, controlled by a GntR family regulator MSMEG_5174 in *M. smegmatis* or its homologous gene Rv1152 in *M. tuberculosis*, manipulates the bacterial susceptibility to aminoglycoside antibiotics. Our study uncovered a mechanism for how bacteria manipulate antibiotics lethality via targeting purine metabolism. Therefore, targeting bacterial purine metabolism may serve as a promising strategy for the treatment of the infectious disease caused by drug-resistant pathologic bacteria, such as *M. tuberculosis*, the causative agent of tuberculosis.

Disturbance in purine metabolism was linked to increased prevalence and progression of many diseases, such as chronic kidney disease (Mazumder et al., 2018), Huntington’s disease (Tomczyk et al., 2021), coronary artery disease (Swain et al., 1982), Alzheimer’s disease (Ansoleaga et al., 2015), and Parkinson’s disease (Garcia-Esparcia et al., 2015). It is plausible to think that dysregulated purine metabolism in these patients could be more susceptible to infection, as the purine metabolites accumulation could decrease the antibiotics killing. Therefore, compounds targeting purine metabolism are being developed for the reconstruction of purine pool that would be a promising strategy for bacterial killing.

## Data Availability Statement

The datasets presented in this study can be found in online repositories. The names of the repository/repositories and accession number(s) can be found below: https://www.ncbi.nlm.nih.gov/bioproject/PRJNA849755.

## Author Contributions

WD, ZZ, SG, and HZ conceived and designed the experiments and wrote the manuscript. WD, ZZ, and YC performed most experiments with assistance from MY, JY, WL, and JZ. WD, ZZ, and JZ analyzed the data. JX modified the manuscript. All authors have read and approved the manuscript.

## Conflict of Interest

The authors declare that the research was conducted in the absence of any commercial or financial relationships that could be construed as a potential conflict of interest.

## Publisher’s Note

All claims expressed in this article are solely those of the authors and do not necessarily represent those of their affiliated organizations, or those of the publisher, the editors and the reviewers. Any product that may be evaluated in this article, or claim that may be made by its manufacturer, is not guaranteed or endorsed by the publisher.

## Supplementary Material

Supplementary Figure 1 The effect of MSMEG_5174 deletion on lethality of other antibiotics. WT and MSMEG_5174 mutants were subjected to antibiotics treatments with indicated concentration of ciprofloxacin **(A)**, chloramphenicol **(B)**, rifampicin **(C)**, and isoniazid **(D)**.
