# Supplementary figures and images for: Deficiency of GntR Family Regulator MSMEG_5174 Promotes Mycobacterium smegmatis Resistance to Aminoglycosides via Manipulating Purine Metabolism
Source: Front Microbiol. 2022 Jul 11;13:919538. doi: 10.3389/fmicb.2022.919538 (PMC9309504; doi:10.3389/fmicb.2022.919538)

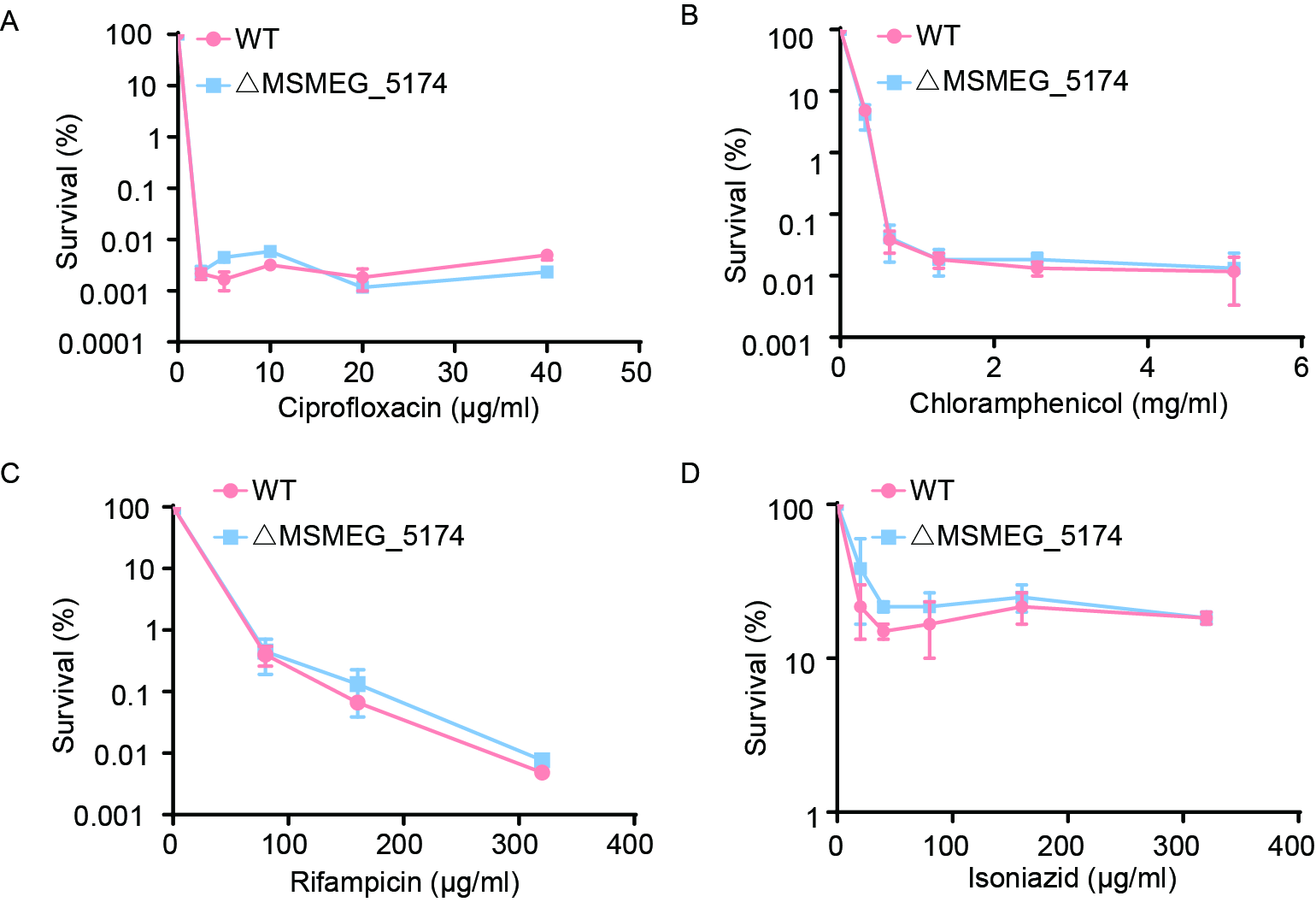

Supplement: Supplementary Figure 1 — The effect of MSMEG_5174 deletion on lethality of other antibiotics. WT and MSMEG_5174 mutants were subjected to antibiotics treatments with indicated concentration of ciprofloxacin (A), chloramphenicol (B), rifampicin (C), and isoniazid (D). [file Image_1.tif]
